# TNF-α- and tumor-induced skeletal muscle atrophy involves sphingolipid metabolism

**DOI:** 10.1186/2044-5040-2-2

**Published:** 2012-01-18

**Authors:** Joffrey De Larichaudy, Alessandra Zufferli, Filippo Serra, Andrea M Isidori, Fabio Naro, Kevin Dessalle, Marine Desgeorges, Monique Piraud, David Cheillan, Hubert Vidal, Etienne Lefai, Georges Némoz

**Affiliations:** 1 Lyon University, INSERM U1060, CarMeN Laboratory, University Lyon-1, Institut National de la Recherche Agronomique UMR1235, INSA-Lyon, F-69600 Oullins, France; 2 Istituto Interuniversitario di Miologia and Dipartimento di Istologia ed Embriologia Medica, Università di Roma-La Sapienza, 00161 Roma, Italy; 3 Dipartimento di Medicina Sperimentale, Università di Roma-La Sapienza, 00161 Roma, Italy; 4 Lyon University, Laboratoire de Physiologie de l'Exercice EA 4338, University Jean Monnet, F-42000 Saint Etienne, France; 5 Hospices Civils de Lyon, Service Maladies Héréditaires du Métabolisme et Dépistage Néonatal, F-69677 Bron, France

## Abstract

**Background:**

Muscle atrophy associated with various pathophysiological conditions represents a major health problem, because of its contribution to the deterioration of patient status and its effect on mortality. Although the involvement of pro-inflammatory cytokines in this process is well recognized, the role of sphingolipid metabolism alterations induced by the cytokines has received little attention.

**Results:**

We addressed this question both *in vitro *using differentiated myotubes treated with TNF-α, and *in vivo *in a murine model of tumor-induced cachexia. Myotube atrophy induced by TNF-α was accompanied by a substantial increase in cell ceramide levels, and could be mimicked by the addition of exogenous ceramides. It could be prevented by the addition of ceramide-synthesis inhibitors that targeted either the *de novo *pathway (myriocin), or the sphingomyelinases (GW4869 and 3-*O*-methylsphingomyelin). In the presence of TNF-α, ceramide-synthesis inhibitors significantly increased protein synthesis and decreased proteolysis. In parallel, they lowered the expression of both the *Atrogin-1 *and *LC3b *genes, involved in muscle protein degradation by proteasome and in autophagic proteolysis, respectively, and increased the proportion of inactive, phosphorylated Foxo3 transcription factor. Furthermore, these inhibitors increased the expression and/or phosphorylation levels of key factors regulating protein metabolism, including phospholipase D, an activator of mammalian target of rapamycin (mTOR), and the mTOR substrates S6K1 and Akt. *In vivo*, C26 carcinoma implantation induced a substantial increase in muscle ceramide, together with drastic muscle atrophy. Treatment of the animals with myriocin reduced the expression of the atrogenes *Foxo3 *and *Atrogin-1*, and partially protected muscle tissue from atrophy.

**Conclusions:**

Ceramide accumulation induced by TNF-α or tumor development participates in the mechanism of muscle-cell atrophy, and sphingolipid metabolism is a logical target for pharmacological or nutritional interventions aiming at preserving muscle mass in pathological situations.

## Background

A major complication arising from a variety of pathological states, including cancer, renal insufficiency, diabetes, and sepsis, is a loss of skeletal muscle tissue that leads to reduced mobility and quality of life, lowered response to treatments, and decreased life expectancy. The causes of the muscle wasting that occurs during chronic diseases are complex, but elevation of pro-inflammatory cytokine levels, in particular TNF-α, is thought to play a prominent role [1]. TNF-α triggers multiple cell responses, including ceramide formation, through stimulation both of a *de novo *synthesis pathway consisting of the condensation of palmitoyl-CoA with serine, and of sphingomyelinase-mediated hydrolysis of membrane sphingomyelin [2,3]. Ceramide is a bioactive mediator involved in cell responses to stress [4]. It is also the central compound of sphingolipid metabolism that gives rise to more complex structural sphingolipids, and to other bioactive mediators such as sphingosine or sphingosine-1-phosphate (S1P) [5]. Whereas the involvement of ceramide in the development of insulin resistance in muscle and of type 2 diabetes has been largely documented [6-8], very little is known about its role in muscle-mass regulation, particularly in muscle atrophy. However, in view of the recognized influence of ceramide on a number of pathways able to affect this tissue, such an involvement would be expected. Ceramide has indeed been shown to inhibit myogenic differentiation [9], amino acid transport, mammalian target of rapamycin (mTOR) activity, and protein synthesis in myotubes [10]. It can also enhance pathways involved in proteolysis, such as the nuclear factor (NF)κB pathway [11] and autophagy [12,13].

We therefore hypothesized that the biosynthesis of sphingolipid mediators, particularly ceramide, participates in the mechanisms leading to muscle loss associated with pathological states. To test this assumption, we used differentiated L6 and C2C12 myotubes treated with TNF-α as *in vitro *models of muscle atrophy, and an *in vivo *mouse model of tumor-induced cachexia [14]. Our results indicate that sphingolipids markedly influence the size and protein metabolism of differentiated myotubes. In parallel, they affect the Akt/mTOR signaling pathway, which is closely involved in the regulation of protein synthesis and degradation [15,16], and phospholipase D (PLD), an activator of this pathway [17,18]. The protective action of the inhibitor of *de novo *sphingolipid synthesis myriocin [19], which we observed both *in vitro *and *in vivo *during tumor-induced cachexia, suggests that preventing ceramide accumulation could represent a promising strategy to preserve muscle mass against the atrophy associated with a number of chronic diseases.

## Results

### Both TNF-α and ceramide induce an *in vitro *atrophy of cultured myotubes

**Figure 1:**
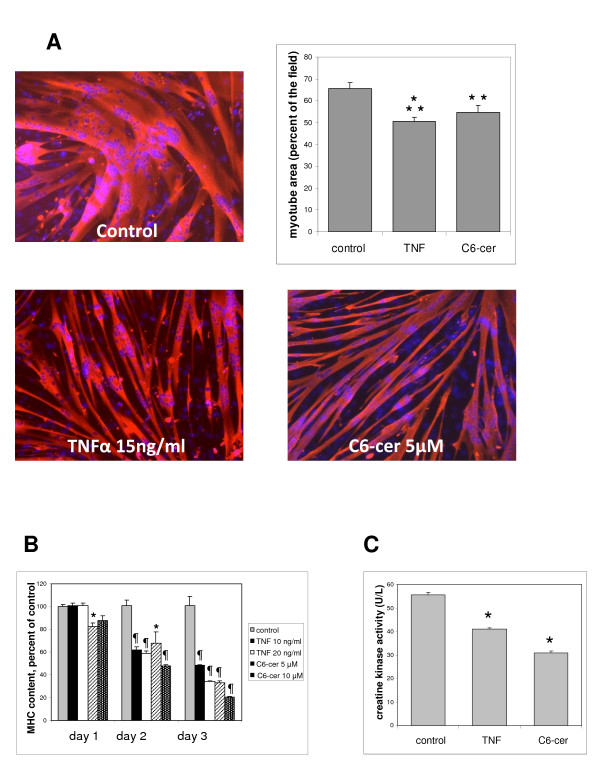
**Effects of tumor necrosis factor (TNF)-α and exogenous ceramide on L6 myotube characteristics**. **(A) **Differentiated myotubes treated for 3 days with 15 ng/ml TNF-α or 5 μmol/l C6 ceramide were immunostained for sarcomeric myosin heavy chain (MHC), and their surfaces were evaluated by using Image J software. The graph shows the myotube area (mean ± SE) expressed as a percentage of the field surface, as determined in ten fields, and is representative of at least three experiments. Different from control: **P = 0.01; ***P = 0.001. **(B) **The MHC content of myotubes treated for 3 consecutive days with TNF-α or C6 ceramide at the indicated concentrations was evaluated by ELISA. The mean ± SE of three measurements expressed as a percentage of control values is shown. The mean control value was 75 ± 11 μg of MHC per 120,000 cells. *Different from control: *P *< 0.05; *P *≤ 0.001. **(C) **The creatine kinase activity of myotubes treated for 3 days with 15 ng/ml TNF-α or 5 μmol/l C6 ceramide was assayed in triplicate (mean ± SE).*Different from control: *P *< 0.05.

In differentiated myotubes of the L6 cell line submitted to 15 ng/ml recombinant TNF-α treatment for 3 days, cell atrophy was present, as evidenced by a significant decrease in cell surface, as already reported [20] (Figure 1a). Other parameters reflecting the functional status of the differentiated muscle cells were also significantly reduced by TNF-α treatment, such as the myosin heavy chain (MHC) content, as evaluated by ELISA (Figure 1b), and the creatine kinase (CK) activity (Figure 1c). Similarly, TNF-α treatment induced a decrease in cell surface in myotubes derived from the C2C12 line (see Additional file 1). We verified that in these conditions TNF-α induced no change in cell viability (not shown). The effects of TNF-α on cellular levels of sphingolipids were assessed in L6 myotubes by tandem mass spectrometry (MS/MS). As expected, TNF-α treatment was able to increase the levels of ceramide, in this case, by 35%. The bulk of the increase mainly concerned a subset of ceramide molecular species: C16:0, C24:1, and C18:0 ceramides (Table 1). TNF-α action also resulted in a 30% decrease in sphingomyelin, especially the C16:0 and C24:1 molecular species, reflecting an activation of sphingomyelinases (Table 1). To assess whether ceramide accumulation could explain the atrophic effects of TNF-α, we investigated the effects of myotube treatment by exogenous ceramide. Interestingly, in both the L6 and C2C12 cell lines, the atrophic effects of TNF-α were mimicked by the addition to the culture medium of cell-permeating short-chain ceramides, particularly C6 ceramide (Figure 1a-c; see Additional file 1), suggesting that myotube atrophy might result from TNF-α-induced ceramide accumulation.

**Table 1:** Effect of tumor necrosis factor (TNF)-α treatment on ceramide and sphingomyelin content of L6 myotubes.^1^

Principal molecular species	Ceramide			Sphingomyelin		
	
	Control, pmol/μg protein	TNF-α, pmol/μg protein	TNF-α-induced increase, % of total control^2^	Control, pmol/μg protein	TNF-α, pmol/μg protein	TNF-α-induced decrease, % of total control^2^
C16:0	4.87 ± 0.82	6.21 ± 1.06	**11.5 ± 5.1**	69.7 ± 10.54	47.2 ± 7.66	**20.4 ± 2.1**

C18:0	1.76 ± 1.11	2.48 ± 0.74	**6.2 ± 2.8**	6.28 ± 0.94	5.35 ± 0.82	**1.1 ± 0.1**

C20:0	0.07 ± 0.07	0.38 ± 0.09	**1.8 ± 0.3**	1.29 ± 0.13	1.12 ± 0.11	**0.16 ± 0.07**

C22:1	0.51 ± 0.15	0.72 ± 0.14	**1.5 ± 0.6**	3.17 ± 0.32	2.77 ± 0.38	**0.54 ± 0.07**

C22:0	0.65 ± 0.18	1.05 ± 0.19	**2.3 ± 0.8**	3.15 ± 0.6	2.62 ± 0.46	**0.55 ± 0.14**

C24:1	4.77 ± 0.91	6.32 ± 1.16	**9.2 ± 2.3**	13.7 ± 2.44	10.00 ± 1.81	**2.72 ± 0.22**

C24:0	1.35 ± 0.33	1.44 ± 0.21	**1.5 ± 0.7**	5.01 ± 1.03	4.39 ± 0.94	**0.55 ± 0.08**

Total	14.70 ± 3.40	19.70 ± 3.85	**35.5 ± 8.8**	118.8 ± 17.2	85.7 ± 13.1	**30.4 ± 2.1**

**^1^**Data are the mean ± SE of five measurements.

^2^TNF-α-induced change in one particular molecular species, expressed as a percentage of the sum of all molecular species in the control.

### Inhibitors of ceramide synthesis prevent TNF-α-induced myotube atrophy

**Figure 2:**
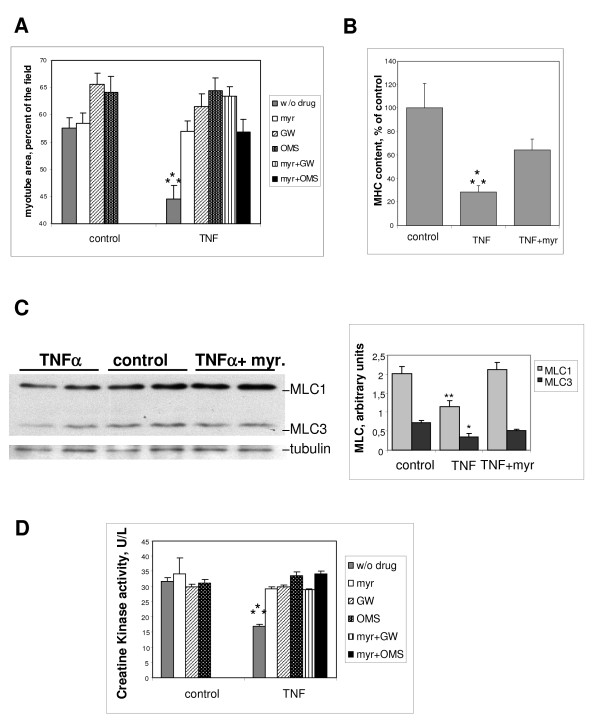
**The negative effects of tumor necrosis factor (TNF)-α on L6 myotubes are counteracted by ceramide-synthesis inhibition**. **(A) **Myotube area was measured after 3 days in the presence or absence of 15 ng/ml TNF-α, with the addition of 100 nmol/l myriocin, 10 μmol/l GW4869, or 1 μmol/l 3-0-methylsphingomyelin (OMS), or with myriocin plus either GW4869 or OMS. Shown are the mean ± SE of 5 to 9 experiments, with 10 fields considered for each condition. ***Different from all the other conditions: *P *≤ 0.001. **(B) **ELISA quantification of the MHC content of myotubes treated for 3 days with 15 ng/ml TNF-α, with or without 100 nmol/l myriocin. The means ± SE of three measurements expressed as a percentage of control values are shown. ***Different from the other conditions: *P *≤ 0.005. **(C) **Western blot analysis of myosin light chains (MLC) 1 and 3 from myotubes treated for 3 days with 15 ng/ml TNF-α, with or without 100 nmol/l myriocin. The diagram shows the quantification of MLC1 and MLC3 levels, after normalization by tubulin levels. Shown are the mean ± SE of three determinations. **Different from control and from TNF-α: *P *≤ 0.002; *different from control: *P *≤ 0.05. **(D) **Creatine kinase activity of myotubes treated or not by TNF-α, in the presence of 100 nmol/l myriocin, or 10 μmol/l GW4869, or 1 μmol/l OMS, or both myriocin and GW4869 or OMS. Shown are the mean ± SE of three determinations made in triplicate. ***Different from all the other conditions: *P *≤ 0.001.

To confirm the role of ceramide formation in the atrophic response to TNF-α, inhibitors of ceramide synthesis were added to the culture medium simultaneously with TNF-α. Ceramide can be formed by two different pathways (under the action of sphingomyelinases or through *de novo *synthesis), and TNF-α is known to activate both pathways [2,3]. Thus, two types of agents were used: myriocin, an inhibitor targeting *de novo *synthesis by selectively inhibiting the first step of the pathway catalyzed by serine-palmitoyl-CoA transferase [21], and GW4869 [22] and 3-O-methylsphingomyelin (OMS) [23], two inhibitors of sphingomyelinases. Myriocin was able to protect L6 myotubes from the TNF-α-induced decrease in surface. GW4869 and OMS were also able to counteract the TNF-α atrophic effect in L6 myotubes, suggesting that ceramide formed by either of the pathways mediates the atrophic effect of TNF-α (Figure 2a). The inhibitors had little influence on myotube surface in the absence of TNF-α, although there was a weak positive effect for GW4869 and OMS. No additive effects of the inhibitors of the two different ceramide-synthesis pathways were seen (Figure 2a).

The effects of ceramide-synthesis inhibition in L6 myotubes were also evaluated using other markers of muscle-cell integrity. Myriocin significantly decreased the TNF-α-induced loss of MHC content as evaluated by ELISA (Figure 2b), and prevented the loss of myosin light chains 1 and 3, as evaluated by western blotting (Figure 2c). Moreover, all of the studied inhibitors of ceramide synthesis had a protective effect against TNF-α-induced CK activity alteration. Again, their actions were not additive (Figure 2d).

In C2C12 myotubes, GW4869 and OMS also showed protective effects against TNF-α-induced atrophy, whereas myriocin was devoid of protective effects, and in fact, produced by itself a negative effect on myotube size, contrary to its effects on L6 myotubes (see Additional file 2). This suggests that, in C2C12 cells, ceramide formed by sphingomyelinase activation is predominant in the induction of atrophy by TNF-α and/or that *de novo *sphingolipid synthesis is necessary for these cells to maintain their homeostasis, by supplying cells with an essential component.

**Figure 3:**
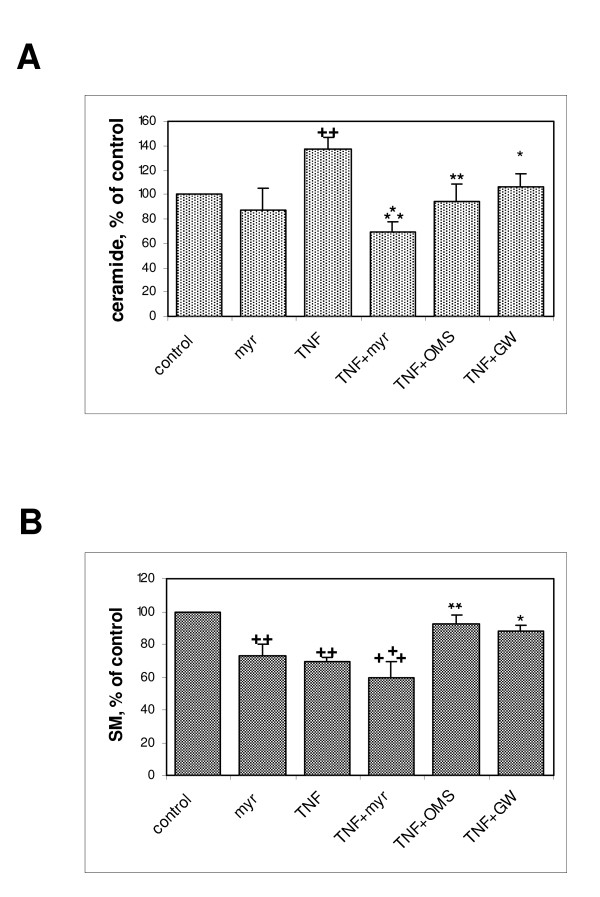
**Inhibitors of ceramide synthesis modulate tumor necrosis factor (TNF)-α effects on myotube sphingolipid content**. **(A) **Total ceramide levels were determined in myotubes treated for 3 days with TNF-α, without or with 100 nmol/l myriocin, 10 μmol/l GW4869, or 1 μmol/l OMS. Results are expressed as a percentage of control value, and are the mean ± SE of 3 to 5 determinations. **++**Different from control: *P *= 0.02; *different from TNF-α alone: *P *= 0.05, **:*P *= 0.02, ***P < 0.001. Mean ± SE control value is 14.7 ± 3.4 pmol ceramide/μg proteins. **(B) **Total sphingomyelin levels were determined in myotubes treated for 3 days with TNF-α, without or with 100 nmol/l myriocin, 10 μmol/l GW4869, or 1 μmol/l OMS. Results are expressed as a percentage of control value, and are the mean ± SE of 3 to 5 determinations. **++**Different from control: *P *= 0.002, **+++***P *< 0.001; *different from TNF-α alone: *P *= 0.05, **P = 0.02. Mean ± SE control value is 118.8 ± 17.2 pmol sphingomyelin/μg proteins.

The effects of ceramide-synthesis inhibitors on the changes in cellular levels of sphingolipids induced by TNF-α were assessed in L6 myotubes. Both the *de novo *synthesis inhibitor myriocin and the sphingomyelinase inhibitors GW4869 and OMS significantly inhibited the TNF-α-induced increase in ceramide levels (Figure 3a), confirming that the drugs were active at the concentrations used, and suggesting that ceramide accumulation results from the activation of both pathways under the action of TNF-α. As expected, treatment with GW4869 and OMS alleviated the TNF-α-induced loss of sphingomyelin (Figure 3b). By contrast, myriocin by itself decreased sphingomyelin levels and amplified the sphingomyelin-lowering effect of TNF-α, in agreement with its reported ability to induce a general depletion of sphingolipids, including sphingomyelin [19].

### Are changes in S1P levels also involved in myotube size regulation?

**Figure 4:**
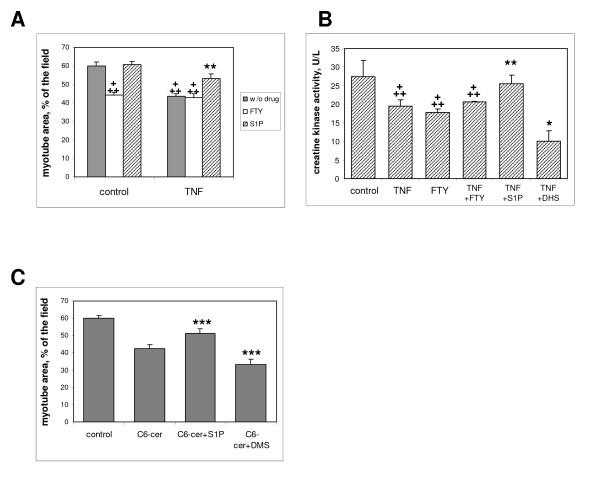
**S1P interferes with the trophic effects of tumor necrosis factor (TNF)-α in L6 myotubes**. **(A) **Myotube area was measured after 3 days of treatment with or without 15 ng/ml TNF-α, in the presence of 1 μmol/l exogenous S1P or 1 μmol/l inhibitor of the S1P receptor FTY720. Shown are the mean ± SE of 10 fields. **+++**Different from control without drug: *P *< 0.0001; **different from TNF-α alone, *P *= 0.004. **(B) **Creatine kinase activity of myotubes treated for 3 days with TNF-α, without or with 1 μmol/l exogenous S1P, 1 μmol/l FTY720, or 10 μmol/l of the sphingosine kinase inhibitor dihydrosphingosine (DHS). Results are shown as the mean ± SE of 3 to 7 experiments performed in triplicate. **+++**Different from control: *P *< 0.003; **different from TNF-α alone: *P *< 0.01, *P < 0.05. **(C) **Myotube area was measured after 3 days of treatment with 5 μmol/l C6 ceramide, without or with 1 μmol/l S1P or 10 μmol/l of the sphingosine kinase inhibitor dimethylsphingosine (DMS). Shown are the mean ± SE of 10 fields. ***Different from C6 ceramide alone: *P *< 0.005.

Because ceramide can be rapidly metabolized in the cell, and potentially converted into the bioactive mediator S1P through the sequential action of ceramidases and sphingosine kinases [5], we evaluated the effects of S1P on myotubes. In L6 myotubes, exogenous S1P in the presence of TNF-α had a positive effect, on myotube surface and on CK activity (Figure 4a, b), suggesting that ceramide metabolization into S1P can induce effects opposite to that of ceramide itself. This antagonistic action was also supported by the observation that S1P also decreased the atrophic effects of ceramide (Figure 4c). Conversely, inhibition of S1P biosynthesis by the addition of the sphingosine kinase inhibitors D-L-threo-dihydro-sphingosine (DHS) and N,N-dimethylsphingosine (DMS) [24] increased the effects of TNF-α and ceramide on myotube surface or CK activity, supporting the assumption that S1P at least partly antagonizes the effects of ceramide (Figure 4b, c). S1P can be secreted and is known to activate a set of specific membrane surface receptors, of which S1P1, S1P2, and S1P3 are expressed in muscle cells [25]. The effects of FTY720, a compound functionally acting as an inhibitor of S1P receptors [26], were thus evaluated. FTY720 itself had negative effects on myotubes, consistent with a positive influence of S1P, but FTY720 did not amplify the negative effects of TNF-α (Figure 4a, b).

### Protein metabolism is altered by ceramide production

**Figure 5:**
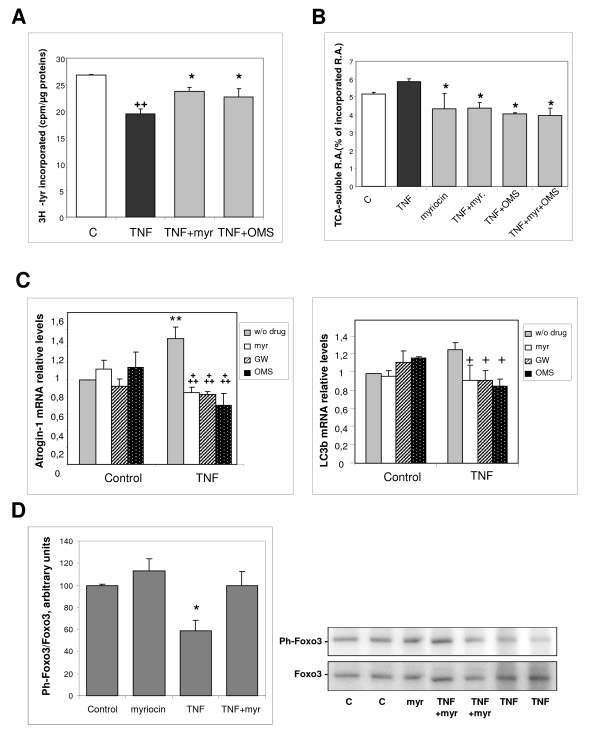
**Effects of tumor necrosis factor (TNF)-α on protein metabolism in L6 myotubes are prevented by ceramide-synthesis inhibition**. **(A) **Myotubes were treated for 12 hours with TNF-α, in the presence or absence of 100 nmol/l myriocin, or 1 μmol/l OMS. The rate of protein synthesis was measured by adding [^3^H]-tyrosine to the culture medium, and counting the radioactivity present in trichloroacetic acid (TCA) precipitates of the cells, in relation to the total protein content. The results are the mean ± SE of three determinations. **++**Different from control: *P *= 0.001; *different from TNF-α alone: *P*≤ 0.05. **(B) **Proteolysis was measured in L6 myotubes labeled with [^3^H]-tyrosine for 48 h, and treated for 12 hours with TNF-α in the presence or absence of 100 nmol/l myriocin, 1 μmol/l OMS, or both. TCA-soluble radioactivity released in the medium was measured and related to the total incorporated radioactivity. The results are the mean ± SE of three determinations. *Different from TNF-α alone: *P *< 0.05. **(C) **The mRNA levels of Atrogin-1 and LC3b were measured by reverse transcription quantitative PCR in myotubes treated or not with TNF-α in the presence of 100 nmol/l myriocin, 10 μmol/l GW4869, or 1 μmol/l OMS, and normalized to the TATA box binding protein mRNA. Results are the mean ± SE of 3 to 5 measurements in duplicate. **Different from control without drug: *P *= 0.01; **+++**different from TNF-α alone: *P*≤ 0.001, **+***P *< 0.05. **(D) **Myotubes were treated for 3 days with or without TNF-α, in the presence of 100 nmol/l myriocin. Phospho- Ser253 Foxo3 in cell extracts was analyzed by western blotting. Results were normalized by total Foxo3 protein amounts. *Different from all other values: *P *< 0.02.

Protein synthesis rate was evaluated in L6 myotubes by measuring the incorporation of [^3^H]-tyrosine into neosynthesized proteins. The marked decrease in protein synthesis induced by a 12-hour TNF-α treatment was significantly counteracted by the addition of either myriocin or OMS (Figure 5a). Proteolysis was also quantified in [^3^H]-tyrosine labeled L6 myotubes, by measuring the release of trichloroacetic acid-soluble radioactivity. In the presence of TNF-α for 12 hours, both myriocin and OMS were able to decrease proteolysis significantly (Figure 5b). These results strongly suggest that ceramide formation negatively influences protein synthesis, whereas it activates proteolysis.

Part of cell proteolysis involves the ubiquitin-proteasome system, and ubiquitin ligases are important components of this system. In muscle, atrophy is often associated with upregulation of a set of genes called atrogenes, including the genes encoding the ubiquitin ligases Atrogin-1 and Murf1, which target muscle-specific proteins [27]. We thus evaluated the effect of ceramide-synthesis inhibitors on ubiquitin ligase mRNA expression, and found that TNF-α increased expression of Atrogin-1, whereas myriocin, GW4869 and OMS markedly decreased its expression, confirming that ceramide formation is able to enhance proteolysis (Figure 5c). By contrast, no significant effect of ceramide inhibition on Murf1 expression was seen (not shown). Because a well-identified target of Atrogin-1 in muscle tissue is the translation initiation factor subunit eIF3f (eukaryotic translation initiation factor 3, subunit f), the degradation of which plays a major role in atrophy [28], we assessed the amount of the eIF3f protein, and found that it was significantly reduced in TNF-α-treated myotubes. Addition of myriocin resulted in a partial reversion of this reduction (see Additional file 3), suggesting that TNF-α-induced, ceramide-dependent, Atrogin-1 upregulation has a negative effect on protein synthesis and myotube size through the degradation of eIF3f, and conversely, that the preservation of eIF3f might participate in the protective effects of ceramide-synthesis inhibition on muscle cells.

Another proteolytic system that is known to contribute to muscle atrophy is autophagy [29], a typical marker of which is LC3b, a protein constituent of autophagosomes [30]. We found that ceramide-synthesis inhibitors significantly decreased the expression of LC3b in the presence of TNF-α, suggesting that ceramide also participates in enhancing proteolysis in myotubes through the autophagic system (Figure 5c). However, ceramide-synthesis inhibitors did not induce significant downregulation of other autophagy marker genes such as *Beclin-1*, *Gabarapl1 *or *CathepsinL *(not shown). It has been reported that both proteasomal and autophagic protein degradation pathways are under the control of the Foxo3 transcription factor, which regulates the expression of atrogenes, including Atrogin-1, and of autophagy-related genes such as LC3b [29]. Foxo transcription factors are negatively regulated by Akt-mediated phosphorylation, which induces their exclusion from the nucleus and thereby inhibits their transcriptional activity [16]. We evaluated the phosphorylation status of Foxo3 in myotubes and, consistent with the induced changes in atrogene expression, TNF-α-treatment caused a significant dephosphorylation, that is, an activation of this factor. This effect was suppressed by myriocin addition, confirming a role of ceramide in TNF-α-induced proteolysis enhancement (Figure 5d).

### Molecular mechanism of ceramide effects on muscle cells

**Figure 6:**
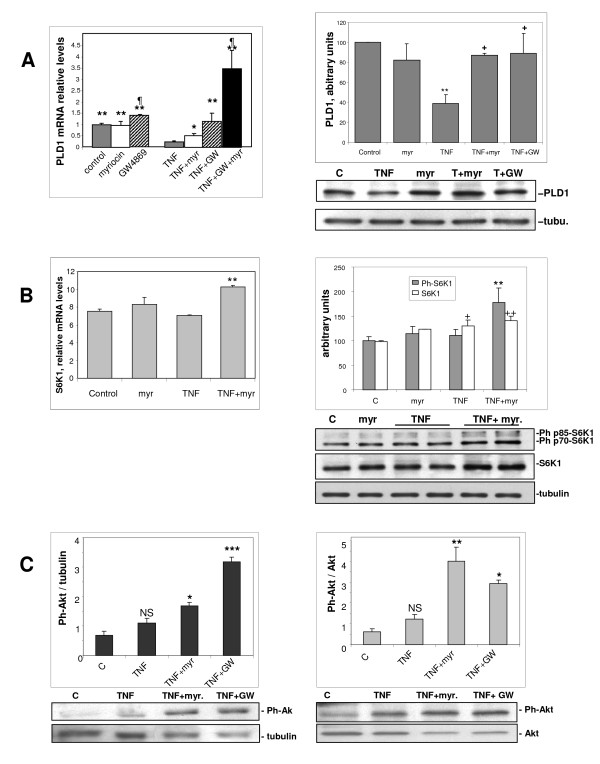
**Inhibitors of ceramide synthesis upregulate anti-atrophic signaling pathways**. **(A) **Myotubes were treated for 3 days with or without tumor necrosis factor (TNF)-α, in the presence of 100 nmol/l myriocin or 10 μmol/l GW4869, or both. (Left panel) Phospholipase (PL)D1 mRNA levels were measured by reverse transcription quantitative PCR, and normalized to the TATA box binding protein mRNA. Results are the mean ± SE of 3 to 6 measurements in duplicate. *Different from TNF-α alone: *P *= 0.05, **P < 0.001; different from control: *P*≤ 0.05. (Right panel) PLD1 protein in cell extracts was analyzed by western blotting. Results were normalized to the amount of tubulin, and are the mean ± SE of three experiments. **Different from control: *P *< 0.01; +different from TNF-α alone: *P *= 0.02. **(B) **Myotubes were treated for 3 days with or without TNF-α, in the presence of 100 nmol/l myriocin. (Left panel) S6K1 mRNA levels were measured by reverse transcription quantitative PCR and normalized to the TATA box binding protein mRNA. Results are the mean ± SE of three measurements in duplicate. **Different from both control and TNF-α alone: *P *< 0.01. (Right panel) Total S6K1 protein and phospho-Thr389 S6K1 in cell extracts were analyzed by western blotting. Results were normalized to the amount of tubulin, and are the mean ± SE of three determinations. +Different from control: *P *< 0.05; ++*P *< 0.02; **different from both control and TNF-α alone: *P *< 0.02. **(C) **Myotubes were treated for 3 days with or without TNF-α, in the presence of 100 nmol/l myriocin or 10 μmol/l GW4869. Ph-Ser473 Akt was analyzed by western blotting. Results were normalized to the amount of (left panel) tubulin or (right panel) total Akt, and are the mean ± SE of 3 to 5 determinations. NS, not significantly different from control. ***Different from TNF-α: *P *< 0.001, **P < 0.01, *P < 0.05.

Ceramide is known to be a downregulator of the expression of PLD, particularly the PLD1 isoform [9,31]. PLD is in turn an activator of mTOR kinase, a major regulator of protein synthesis and degradation [15], closely involved in muscle-tissue homeostasis [32]. We therefore assessed the influence of TNF-α and ceramide-synthesis inhibitors on the expression of PLD1 in L6 myotubes. Myriocin by itself had no influence on PLD1 mRNA, whereas GW4868 alone moderately increased the PLD1 mRNA level. TNF-α strongly repressed the expression of PLD1 mRNA, and ceramide-synthesis inhibitors rescued its expression, with myriocin resulting in partial, and GW4869 in total rescue. In fact, potentiation of the effects of the two inhibitors on PLD1 mRNA levels was seen (Figure 6a). These effects were confirmed at the protein level, with TNF-α inducing a marked decrease in PLD1 protein, which was rescued by either myriocin or GW4869 (Figure 6a). These results thus indicated that TNF-α lowers PLD1 expression in myotubes through the production of ceramide both by the *de novo *pathway and by sphingomyelinase activation.

It could be expected that the observed changes in PLD1 expression induced by TNF-α, and their reversion by ceramide-synthesis inhibitors, directly influenced the activity of mTOR kinase, an important regulator of protein metabolism. Indeed, we recently found that phospholipase D is able to activate both protein complexes involving mTOR kinase (mTORC1 and mTORC2) in L6 myoblasts [17]. A well-identified effector of mTORC1 that positively regulates protein translation is S6 kinase (S6K)1 [15]. We evaluated by western blotting the phosphorylation of S6K1 on the Thr389 residue, which reflects its activation state. Contrary to what we expected, we found that S6K1 phosphorylation was barely affected by TNF-α alone; however, it was markedly increased by myriocin addition in the presence of TNF-α (+80%). Under the same conditions, total S6K1 protein was also increased, by 40% (Figure 6b, right panel). This increase was paralleled by a 36% increase in S6K1 mRNA, as evaluated by reverse transcription quantitative PCR (Figure 6b, left panel). It can thus be inferred that, in the presence of TNF-α, ceramide-synthesis inhibition affects protein synthesis by increasing both the expression and the phosphorylation of the mTORC1 effector S6K1.

Akt kinase is a mTORC2 substrate that plays a major role in the control of proteolysis [16]. We thus evaluated the phosphorylation of Akt on Ser473 residues, a reflection of its activation state. Here again, there was an unexpected finding, namely that TNF-α alone tended to have a positive (although not significant) effect, rather than a negative one, on Akt phosphorylation. This positive effect was markedly amplified in the presence of either myriocin or GW4869, with Akt activation being significant whether phospho-Akt was normalized by the amount of tubulin in the samples, or by the amount of total Akt protein (Figure 6c). Thus, in the presence of TNF-α, activation of the mTORC2 substrate Akt is likely to participate in the decrease in proteolysis induced by ceramide-synthesis inhibition.

Together, these data indicated that in L6 myotubes, PLD1 upregulation induced by ceramide-synthesis inhibition in the presence of TNF-α is related to the upregulation and activation of the well-known anabolic factors S6K1 and Akt.

Because TNF-α is well known as an activator of the NFκB pathway [2] that can activate protein catabolism [33], we further investigated the effect of ceramide-synthesis inhibition by myriocin on the phosphorylation of NFκB inhibitor kinase subunit-α/β (IKK-α/β), an essential step of the NFκB activation cascade [33]. We found that TNF-α treatment of L6 myotubes was able to rapidly induce IKKα/β phosphorylation, reaching a maximum in 30 minutes. Notably, myriocin treatment had no influence on this response, suggesting that sphingolipid metabolism was not involved in the activation of NFκB pathway in our setting (see Additional file 4).

### *In vivo *inhibition of ceramide synthesis protects mice against tumor-induced muscle atrophy

**Figure 7:**
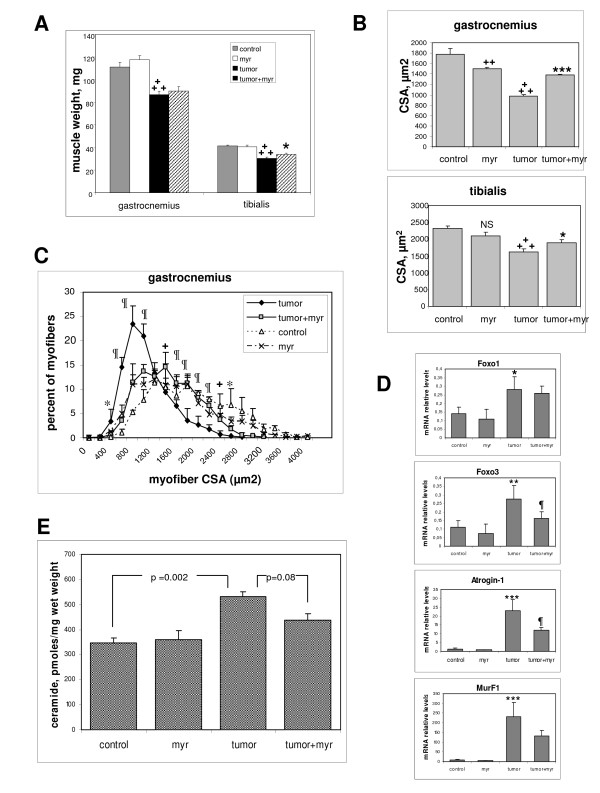
**Myriocin protects muscle tissue from tumor-induced atrophy**. **(A) **Mice were inoculated or not with C26 adenocarcinoma, and were injected daily or not with 0.1 mg/kg myriocin. The gastrocnemius and tibialis muscles were dissected and weighed (mean ± SE of 5 to 7 experiments for gastrocnemius; 7 to 10 for tibialis). **+++**Different from control: *P *< 0.0001; *different from 'tumor': *P *= 0.05. **(B) **Mean cross-sectional area (CSA) of myofibers was evaluated on transverse sections of gastrocnemius and tibialis muscles (mean ± SE of four experiments for gastrocnemius, seven for tibialis). **+++**Different from control: *P *< 0.0001, **++***P *< 0.01; ***different from 'tumor': *P *= 0.0002, *P = 0.05. **(C) **Frequency distribution of myofiber CSA in gastrocnemius (mean ± SE of four experiments). 'Tumor' different from 'tumor+myriocin', *P *< 0.002, **+***P *< 0.01, *P < 0.05. **(D) **mRNA expression of atrophy-related genes in gastrocnemius was evaluated by reverse transcription quantitative PCR, and normalized to cyclophilin A mRNA. Results are the mean ± SE of four determinations in duplicate. *Different from control, *P *< 0.05; **P < 0.01; ***P < 0.001; different from 'tumor', *P *< 0.05. **(E) **Total ceramide levels were measured in the tibialis of the differently treated mice (mean ± SE, four experiments).

The development of C26 tumors in the mice is known to induce severe cachexia, characterized by a rapid loss of muscle mass [14]. Cancer-induced muscle wasting is thought to be related to strongly increased circulating levels of pro-inflammatory cytokines, particularly TNF-α [34]. To evaluate the protective potentialities of ceramide-synthesis inhibition against muscle wasting, we treated C26-bearing mice with myriocin, a drug that has the ability to decrease muscle ceramide levels *in vivo *[8]. The development of the C26 tumor induced a rapid fall in the animals' weight after 10-15 days, confirming the occurrence of cachexia. Tumor-induced muscle atrophy was assessed by measuring the weights of the gastrocnemius and tibialis anterior muscles, and the cross-sectional area (CSA) of myofibers in these muscles. Under the effect of tumor growth, the gastrocnemius and tibialis weights were decreased by 22% and 26%, respectively (Figure 7a), and the mean CSA in these muscles was reduced by 45% and 31%, respectively (Figure 7b). Myriocin treatment had no effect on the timing of cachexia onset or on tumor size (not shown), but it did tend to increase the weight of the hindlimb skeletal muscles in the presence of tumor, with a significant increase of +11% in the tibialis muscle (Figure 7a). In control mice, myriocin treatment alone had a small negative effect on mean CSA in both muscles ((-16% in gastrocnemius (significant) -10% in tibialis (non-significant)), but in the presence of tumor, myriocin treatment significantly counteracted the CSA decrease (Figure 7b).Taking into account the size distribution of myofibers in gastrocnemius, the presence of tumor was clearly associated with the disappearance of the largest fiber population, and myriocin restored the presence of large fibers at the expense of the small-fiber population (Figure 7c).

The atrophic effect of the tumor was also illustrated by a large increase in the expression of the *Atrogin-1 *and *MurF1 *atrogenes, and a significant increase in the expression of the Foxo1 and Foxo3 transcription factors, which are positive regulators of atrogene expression [16]. Treatment of tumor-bearing mice with myriocin significantly decreased expression of Atrogin-1 and Foxo3 (Figure 7d).

To evaluate whether ceramide levels were altered in muscle tissue under the different conditions, ceramide was quantified in the tibialis muscles of the same mice. In agreement with our assumption that ceramide is involved in atrophy, the tumor induced a marked increase in muscle ceramide level (+53%). Myriocin treatment of tumor-bearing mice lowered ceramide levels, although the difference did not reach statistical significance (Figure 7e). By contrast, no significant variations in sphingomyelin levels were detected (not shown).

Together, these observations show that blocking *de novo *ceramide synthesis has an anti-atrophic effect *in vivo *in tumor-bearing mice.

## Discussion

In this study, we found that *in vitro *TNF-α treatment was able to significantly decrease the surface area of myotubes deriving from either the L6 or the C2C12 lines, with this effect being reproduced by addition of cell-permeating ceramides. Furthermore, both TNF-α and ceramide decreased the CK activity and MHC content of L6 myotubes. These observations suggested that the atrophic effects of TNF-α on muscle cells might rely on the production of ceramide triggered by the cytokine.

To verify this hypothesis, we used different ceramide-synthesis inhibitors together with TNF-α. Both a *de novo *pathway inhibitor (myriocin) and two sphingomyelinase inhibitors (GW4869 and OMS) were able to suppress the effects of TNF-α on L6 myotube size, myosin heavy and light chain content, and CK activity, suggesting that ceramide formed by either of the pathways mediates the atrophic effects of TNF-α. The effects of inhibitors of the two different ceramide-synthesis pathways were not additive, suggesting that a mere reduction in ceramide formation, rather than complete suppression, is enough to prevent cell atrophy. Alternatively, this could result from the overall depletion of sphingolipids induced by the blockade of *de novo *synthesis by myriocin [19], which potentially also reduced sphingomyelinase-mediated ceramide synthesis. The sphingomyelinase inhibitors GW4869 and OMS also showed protective effects against TNF-α-induced atrophy in C2C12 myotubes, thereby confirming the involvement of ceramide formed by sphingomyelinase activation. However, in this cell line, myriocin was devoid of protective effects and in fact, showed a negative effect by itself on myotube size, contrary to the results in L6 myotubes. A possible explanation is that in the C2C12 line *de novo *sphingolipid synthesis has to be maintained above a certain threshold, so as to supply cells with a compound that can be either a structural component such as sphingomyelin or a glycosylceramide, or a mediator such as S1P, and this would be essential to maintain cell homeostasis.

Quantification of the sphingolipids established that TNF-α markedly increased the ceramide content of L6 myotubes, consistent with a role for this sphingolipid mediator in the atrophic response. Ceramide accumulation was accompanied by a substantial decrease in sphingomyelin cell content, showing that ceramide resulted, at least in part, from sphingomyelin hydrolysis. However, among the molecular species of ceramide formed under TNF-α stimulation, the C18:0 species was found to be one of the most important (its increase representing 6.2% of the sum of ceramide species in the control), whereas there was no parallel hydrolysis of C18:0-sphingomyelin (Table 1). It can thus be hypothesized that TNF-α was able to induce *de novo *synthesis of C18:0 ceramide, and to produce other species, mainly C16:0 and C24:1 ceramides, via sphingomyelinase activation. Myriocin totally prevented the TNF-α-induced rise in ceramide, and in parallel decreased the levels of sphingomyelin, supporting the above proposal that it is able to inhibit both the *de novo *and the sphingomyelinase pathways of ceramide synthesis. Similar to myriocin, the sphingomyelinase inhibitors were able to diminish TNF-α-induced ceramide rise, although to a lesser extent, and as expected, these inhibitors significantly restored the sphingomyelin cellular content. On the whole, these data are compatible with the interpretation that myotube atrophy induced by TNF-α involves accumulation of ceramide, and that the inhibitors counteracted the TNF-α effects by preventing ceramide synthesis.

Because ceramide can be rapidly metabolized and give rise to other mediators, of which S1P has particular importance in cell physiology [5], we considered the possibility that this ceramide metabolite could interfere in the response of L6 myotubes to TNF-α stimulation. We found that S1P displayed a positive influence on myotube integrity, opposite to that of exogenous ceramide or TNF-α. A lowering of S1P availability might thus participate in the negative effects of TNF-α. Consistent with this idea, these effects were not amplified by S1P receptor inhibition, suggesting that S1P levels had already been reduced below an effective concentration by TNF-α treatment. These observations are in agreement with the reported attenuation of denervation-induced soleus atrophy by S1P perfusion in the rat [35].

It is well known that muscle atrophy results from both decreased protein synthesis and accelerated proteolysis. In agreement with a pro-atrophic role of TNF-α, we found that the cytokine lowered protein synthesis and tended to increase proteolysis in L6 myotubes. These effects were reversed by the ceramide-synthesis inhibitors myriocin and OMS, indicating that ceramide is involved in the effects of TNF-α on protein metabolism. Concerning the elements of the proteolytic machinery that are regulated by ceramide, we could observe that ceramide-synthesis inhibition markedly lowered the expression of Atrogin-1 ubiquitin ligase, which was increased by TNF-α. As this atrogene plays a recognized role in the catabolism of muscle-specific proteins, at least part of the TNF-α/ceramide atrophic effects can be related to the modulation of this factor, and to the resulting decrease in eIF3f translation initiation factor levels. In addition, we found that ceramide-synthesis inhibitors significantly lowered the expression of LC3b, an essential component of the autophagic proteolytic system. The activation of autophagy by ceramide has already been described in other cell systems [13]. It is thus possible that ceramide positively regulates both proteasome- and autophagy-dependent protein degradation in differentiated myotubes, possibly through coordinated changes in Foxo3 transcription factor phosphorylation. However, the transcriptional regulation of autophagy by ceramide in L6 myotubes remains speculative, in view of the absence of effect of ceramide-synthesis inhibitors on the expression of several other autophagy-related genes.

Protein homeostasis is, for a large part, under the control of signaling pathways of which the central elements are the kinases Akt and mTOR. These interconnected regulators integrate inputs from growth factors, nutrient availability, and energy levels so as to adapt protein synthesis and degradation to the physiological status of the cell. Akt is a major inhibitor of proteolysis through the control of Foxo transcription factors, which in turn regulate the expression of ubiquitin ligases involved in the specific degradation of muscle proteins by proteasome [16]. mTOR kinase, in complex with the protein Raptor (mTORC1 complex), is indirectly activated by Akt, through phosphorylation of its inhibitor tuberous sclerosis complex [15]. Activated mTORC1 is known to enhance protein translation, particularly through the activation of its substrate S6K1, and has been shown to be required for muscle hypertrophy [36]. mTORC1 is also a major negative regulator of autophagy [15]. Furthermore, mTOR also exists in complex with the protein Rictor to form the mTORC2 complex, which is able to phosphorylate and activate Akt. Both mTOR complexes are stimulated by the phospholipid messenger phosphatidic acid [17,18], the product of the action of the signaling enzyme of cell membranes, PLD. Moreover, involvement of PLD in mTOR activation in response to exercise has been shown, suggesting its role in muscle-tissue hypertrophy [37].

Ceramide is considered a general inhibitor of PLD, acting at the catalytic site, on the recruitment of activator proteins, and also at the transcriptional level [38]. We previously found that ceramide selectively inhibits expression of the PLD1 isoform of the enzyme in L6 myoblasts [9]. In the present study, we found that TNF-α markedly decreased expression of PLD1, and that ceramide-synthesis inhibitors rescued its expression, suggesting that PLD1 is one major target of ceramide in this signaling network. Because PLD is an activator of both mTOR complexes, we then considered the influence of these inhibitors on the mTORC1 substrate S6K1 and the mTORC2 substrate Akt. Ceramide inhibition in the presence of TNF-α increased the amounts of both S6K1 and Akt under the phosphorylated/activated state, possibly as a consequence of PLD1 upregulation. However, a discrepancy between PLD1 expression and S6K1/Akt phosphorylation state was apparent under the effect of TNF-α alone, which downregulated PLD1 without affecting, or even slightly enhancing, either S6K1 or Akt phosphorylation. A possible explanation for this is that the pleiotropic cytokine TNF-α may trigger other signaling pathways that are able to positively influence Akt and mTORC1, and thereby mask any negative effects of ceramide on S6K1 and Akt. By suppressing these negative effects, ceramide-synthesis inhibition would allow further activation of these mTOR effectors. Inhibition of TNF-α-induced ceramide accumulation could thus have positive trophic effects on muscle cells, at least partly through the upregulation of PLD1 and the resulting activation of S6K1 and Akt, which respectively enhance protein synthesis and reduce proteolysis. However, TNF-α by itself altered protein synthesis without having significant effects on S6K1 and Akt, thus we hypothesize that the cytokine triggered other undefined mTOR-independent pathways that negatively influenced proteosynthesis.

Another mechanism by which ceramide-synthesis inhibition might result in Akt stimulation and positive effects on myocyte size is related to the recognized ability of ceramide to hamper insulin/insulin-like growth factor (IGF) signaling in muscle tissue [6,39]. Myriocin has thus been shown to decrease muscle ceramide levels and insulin resistance in mice placed on a high-fat diet [7,8]. Because IGFs are involved in trophic effects on muscle tissue [40], it is possible that in our study myriocin acted on myocyte size, both *in vitro *and *in vivo*, through the enhancement of signaling by endogenously produced IGF, and downstream Akt activation. However, we found no changes in IRS-1 tyrosine phosphorylation under TNF-α and myriocin treatments of L6 myotubes (not shown), which makes a role for IGF signaling in the effects of myriocin very unlikely. In addition, our *in vitro *results suggest that myriocin does not modulate proteolysis by targeting the NFκB pathway, and thus that this pathway is not regulated by ceramide in muscle cells.

Finally, our study addressed the *in vivo *role of ceramide in a model of tumor-induced cachexia. The development of C26 adenocarcinoma induced a marked increase in ceramide levels in mouse muscle, together with severe atrophy. A low dose of myriocin (0.1 mg/kg) significantly limited muscle loss, reduced expression of some atrogenes, and partially restored myocyte size, confirming that ceramide accumulation participates in enhanced proteolysis and muscle atrophy. As for the small negative effect of myriocin alone on myocyte size, it might be attributable, similarly to the hypothesis for C2C12 cells, to a lowered supply in sphingolipid(s) involved in the maintenance of muscle-tissue homeostasis.

## Conclusions

This study has established that inhibition of ceramide synthesis has beneficial effects on myocyte size under conditions inducing muscle atrophy. The sphingolipid pathway thus could be a possible target of interventions aiming at protecting muscle tissue against the wasting that occurs in various pathological situations, particularly during cancer-induced cachexia. The therapeutic potential of inhibitors of *de novo *ceramide synthesis and of sphingomyelinase action thus deserves further investigation. In addition, because ceramide synthesis depends on cell availability in palmitic acid, and can be altered by the composition of fatty acid dietary intake [41,42], it will be of interest to consider nutritional interventions targeting the sphingolipid pathway in the treatment of muscle atrophy.

## Methods

GW4869 was from Sigma-Aldrich (L'Isle-d'abeau, France). Sphingosine-1-phosphate, DHS, and 3-OMS were obtained from Enzo Life Sciences (Paris, France). C6 ceramide and N,N-dimethylsphingosine were from Biomol Research Laboratories Inc., (Plymouth Meeting, PA, USA). FTY 720 was from Cayman Chemical Company (Ann Arbor, MI, USA). Anti-phospho-Thr389/Thr412-S6K1 antibody, anti-S6K1 antibody, anti-phospho-Ser473-Akt antibody, anti-Akt antibody, anti-phospho-Ser176/180-IKKα/β and anti-PLD1 polyclonal antibody were from Cell Signaling Technology (Beverly, MA, USA). Anti-eIF3f antibody was from Rockland (Gilbertsville, PA, USA). Anti-Foxo3 and anti-phospho-Ser253-Foxo3 antibodies were from Millipore (Billerica, MA, USA). Anti-α-Tubulin monoclonal antibody was supplied by Sigma. Anti-myosin light chain 1 and 3 monoclonal F310 antibody and anti-sarcomeric MHC MF-20 antibody were from the Developmental Studies Hybridoma Bank, University of Iowa (Iowa City, USA). HRP-conjugated anti-rabbit-IgG antibodies were from Jackson Immunoresearch Laboratories (Soham, UK).

### Cell culture

L6 myoblasts of the C5 subclone were maintained in Dulbecco's modified Eagle's medium (DMEM) with 4.5 g/l glucose, supplemented with 10% (v/v) fetal bovine serum at 37°C and 5% CO_2_. To induce differentiation, cells were seeded at a density of 5.10^5 ^cells per well in six-well plates, and cultured in DMEM supplemented with 1% fetal bovine serum and 10^-7 ^mol/l arginine-vasopressin (AVP; Sigma-Aldrich) for 5 days. The myotubes obtained were then treated with 15 ng/mL recombinant rat TNF-α (Immunotools, Friesoythe, Germany). for 3 days to induce atrophy, in the presence or absence of sphingolipid synthesis inhibitors. The effect of TNF-α on cell viability was determined by 3-(4,5-dimethyl-2 thiazoyl)-2,5-diphenyl-2H-tetrazolium bromide (MTT) assay (Roche Diagnostics, Meylan, France) in 96-well plates. At the end of TNF-α treatment of differentiated myotubes, they were supplemented with 0.5 mg/mL MTT for 4 h at 37°C. After this incubation period, purple formazan salt crystals formed, and were dissolved in solubilization solution overnight. Optical density was measured at 550 and 690 nm, using an ELISA plate reader (PowerWave X, Biotek Instruments, Winooski, VT, USA). The number of viable cells directly correlated with the difference between the 550 and 690 nm absorbance results.

### Electrophoresis and western blotting

Cells were lyzed in ice-cold buffer containing 20 mmol/l Tris-HCl, 100 mM NaCl, 10 mmol/l sodium pyrophosphate, 10 mmol/l glycerophosphate, 50 mmol/l sodium fluoride, 1.5 mmol/l Na_3_VO_4_, 1% Triton, and a protease inhibitor cocktail (pH 7.6). Lysates were kept on ice during 15 minutes and separated by centrifugation at 13,000 *g *for 15 minutes. Bradford protein assay (Bio-Rad, Marnes-La-Coquette, France) was performed on an aliquot of the solution. Cell lysates were analyzed by SDS-PAGE, and proteins were transferred onto PVDF membranes blocked with 5% BSA in Tris-buffered saline/0.1% Tween 20, and incubated with the various antibodies (see Materials) following the manufacturers' recommendations. Immunoblots were visualized with the ECL detection system (Pierce Biotechnology Inc., Rockford, Illinois, USA) and quantified with Image J software http://rsb.info.nih.gov/ij/. SDS-PAGE was performed using 10% polyacrylamide gels for S6K1, Akt and IKKα/β, and 12% polyacrylamide gels for myosin light chains. In the case of PLD1, the protein was immunoprecipitated before electrophoresis. The cell lysate was incubated with capture anti-PLD1 polyclonal antibody (Cell Signaling Technology) overnight at 4°C with continuous mixing. Protein G magnetic beads (PureProteome Protein G Magnetic Beads Kit; Millipore, Billerica, MA, USA) were pre-washed with PBS (phosphate-buffered saline) containing 0.1% Tween 20, and 50 μL of these were added to the preformed antibody-antigen complex, then the suspension was incubated for 30 min at 4°C. The beads were collected with a magnet, washed in PBS/0.1% Tween, and the elution was performed with Laëmmli buffer 1:3 in lysis buffer. The eluate was heated at 70-90°C for precisely 2 min (to prevent PLD1 aggregation known to occur in overheating conditions). Samples were subjected to SDS-PAGE on 8% acrylamide gels, in the presence of 4 mol/l urea, then transferred to PVDF membrane, and western blotting was performed with anti-PLD1 monoclonal antibody (H00005337-M02; Abnova, Taipei, Taiwan)) diluted 1:500 in TBS/0.1% Tween 20.

### Measurement of sphingolipid levels

Sphingolipids were extracted by the Bligh and Dyer method [43]. Muscle tissue was homogenized (Precellys 24 Homogenizer; Bertin Technologies, Montigny-le-Bretonneux, France) in chloroform:methanol (1:2 v/v) using 1 ml of solvent per 10 mg of wet tissue, in a. Myotubes were scraped into 500 μl 0.1 N HCl in PBS, and 2 mL chloroform/methanol (1:2 v/v) were added. An aliquot of the mixture was kept for Bradford protein assay. Internal deuterium-labeled standards were added at this step: C16:0 D31 ceramide (N-palmitoyl(d31)-D-erythro-sphingosine), and C16:0 D31 sphingomyelin (N-palmitoyl(d31)-D-erythro-sphingosylphosphorylcholine) (Avanti Polar Lipids Inc., Alabaster, AL, USA). The samples were mixed by vortex for 30 seconds, sonicated, and let to stand for 30 minutes at room temperature. One volume of water and one volume of chloroform were added to perform phase separation. The samples were then vortexed again for 1 minute before centrifugation at 750 g for 5 minutes. The lower organic phase was transferred to a clean collection tube, and evaporated under a nitrogen stream at 37°C. The dry extracts were kept at -20°C until analysis by electrospray tandem mass spectrometry (ESI MS/MS), carried out on a triple-quadrupole mass spectrometer (API 3200 MS/MS; Sciex Applied Biosystems, Toronto, Canada) equipped with an ion spray source (TurboIonSpray; Applied Biosystems, Foster City, CA, USA) heated to 500°C. Two binary pumps, a vacuum degasser and a high-performance autosampler (Agilent 1200 Series; Agilent Technologies, Massy, France) with a control module (Instant Pilot; Agilent Technologies) were used for solvent delivery and automated sample introduction. All results were acquired with Analyst software (version 1.5; AB Sciex LLC, Foster City, CA, USA). For measurement of sphingomyelin, the MS/MS was performed in negative ionization mode. Specific parameters used were as follows: ion spray voltage - 4500 V; entrance potential -9.5 V; declustering potential -120 V; collision energy -100 eV. For the measurement of ceramide, the MS/MS was performed in positive ionization mode. Specific parameters used were as follows (in arbitrary units) ion spray voltage 5500 V; entrance potential -10 V; declustering potential 100 V; collision energy 40 eV. Sample analysis for quantification was performed in multiple reaction monitoring (MRM) mode by flow injection analysis (for specific MRM values corresponding to each ceramide or sphingomyelin species and to the corresponding internal standard, see Additional file 5). The dwell time was fixed to 30 ms for each species. Sphingomyelin and ceramide were measured separately, with two different acquisition methods and two different injections (20 μl and 5 μl, respectively), with a flow rate of 200 μl/min 2:1 chloroform:methanol (analysis time of 3 minutes). The concentration of each molecular species was calculated from the ratio of its signal to that of the corresponding internal standard. Total ceramide and sphingomyelin concentrations were the sum of the concentrations of the various species.

### Measurement of the area of immunofluorescence-labeled myotubes

The differentiated myotubes were fixed with 3.7% formaldehyde for 20 minutes at room temperature and permeabilized with 0.1% Triton for 10 minutes at room temperature, then aspecific labeling was blocked with 1% BSA for 20 minutes. Anti-myosin MF-20 antibody was added diluted 1:50 and incubated for 1 hour at room temperature. After washing with PBS/1% BSA, rhodamine-conjugated anti-mouse IgG antibody was added diluted 1:500 in 1% BSA for 1 hour at room temperature. Nuclei were stained with 1 μg/mL 4.5-diamidino-2-phenylindole (DAPI) for 3 minutes. The cells were examined by immunofluorescence microscopy (Axiovert 200 microscope, with an objective LD-A plan 20x/0.30 PHI ∞/40, an Axiocam MRM camera and Axiovision 4.1 image-acquisition software; Carl Zeiss, Göttingen, Germany). Differentiated myotubes, but not myoblasts, were evenly labeled on their entire surface. Their area was measured by the method of Sultan *et al. *[44], using Image J software.

### Assay of creatine kinase activity

Cells were seeded at a density of 5.10^5 ^cells per well in six- well plates and scraped into 500 μl of ice-cold lysis buffer. The assay was performed using a commercial kit (CK-NAC LD B Kit; Sobioda, Montbonnot-St Martin, France) which allows monitoring at 340 nm of the kinetics of formation of NADPH, produced by an enzymatic cascade initiated by CK. The assay was performed in 96-well plates, with 4 μL of sample per well, for 20 minutes at 30°C.

### ELISA of myosin heavy chain

The cells were grown in 12-well plates at a density of 3.10^4 ^cells per cm^2^. After completion of differentiation and 3 days of treatment with TNF-α and various sphingolipid synthesis inhibitors, the cells were scraped into 300 μL ice-cold RIPA buffer, mixed by vortex and separated by centrifugation at 10,000 *g *for 10 minutes. The assay was carried out in 96-well plates. Samples (50 μL)were evaporated to dryness overnight at 37°C, then the wells were washed twice with cold PBS. All washing steps were performed using an automatic plate washer (ELx50 Autostrip Washer from Bio-Tek Instruments, Inc.). Aspecific binding sites were saturated with 100 μL of 0.1% BSA in PBS for 30 minutes at 37°C. Samples were then incubated with 50 μL MF-20 antibody, diluted 1:100 in PBS, for 1 hour at 37°C. After another washing step, samples were incubated with 50 μL of secondary HRP-conjugated anti-mouse antibody (Jackson Immunoresearch Laboratories, Soham, UK) diluted 1:3000, for 1 hour at 37°C. Plates were washed five times, then 50 μL of TMB substrate (Sigma-Aldrich) were added to each well, and 0.5 N H_2_SO_4 _was added after 5 minutes to stop the color reaction. Optical density was read at 450 nm. A standard curve was obtained with purified MHC (Sigma-Aldrich).

### Measurement of protein synthesis

Protein synthesis rates were assayed according to Gulve and Dice [45], with some modifications. Myotubes were treated for 12 hours with TNF-α and various inhibitors. Culture medium was then replaced by experimental medium containing 2 μCi/mL of [^3^H]-L-Tyrosine (Perkin-Elmer, Waltham, MA, USA) and non-radioactive tyrosine up to 2 mol/l and incubated for 1 hour. Culture monolayer was washed five times with ice-cold PBS. Cells were scraped into 10% trichloroacetic acid (TCA) on ice, mixed by vortex and separated by centrifugation at 12,000 *g *for 10 minutes to separate the pellet containing labeled neosynthesized proteins from the supernatant containing the pool of non-incorporated [^3^H]-L-Tyrosine. The pellet was then dissolved in cell lysis buffer and neutralized with 1 mol/l NaOH. An aliquot was taken for Bradford protein analysis. Determination of radioactivity was performed in a liquid-scintillation spectrometer (Hewlett-Packard, Palo Alto, CA, USA).

### Measurement of protein degradation

Rates of protein degradation were determined by monitoring the release of TCA-soluble radioactivity in the culture medium at defined time, after radiolabeling proteins with [^3^H]-L-tyrosine. Long-lived proteins were radiolabeled by incubating the cells with 2 μCi/mL of [^3^H]-L-Tyrosine under differentiating conditions for 2 days, until completion of differentiation. Cells were rinsed three times with PBS and shifted for 2 hours in chase medium (DMEM 1% fetal bovine serum and non-radioactive tyrosine to prevent reincorporation of [^3^H]-L-Tyrosine) to allow degradation of short-lived proteins. Cells were washed again, and the medium replaced. After 12 hours, samples (0.5 mL) of the culture medium were taken and supplemented with 10% (w/v) TCA. After standing at 4°C for 1 h, samples were separated by centrifugation at 12,000 *g *for 10 minutes, to separate the pellet of precipitated proteins from the free amino acids resulting from protein degradation. The pellet was dissolved with 0.5 mL lysis buffer and neutralized with 1 mol/l NaOH. The monolayer was washed three times with PBS and scraped into lysis buffer to determine cell-associated radioactivity. Proteolysis was evaluated as the percentage of TCA-soluble radioactivity released in the medium relative to the total incorporated radioactivity.

### Reverse transcription and real-time PCR

Total RNA was isolated from L6 myotubes using Trizol reagent (Invitrogen Corp., Carlsbad, CA, USA). Total RNA (1 μg) was used for reverse transcription, in the presence of 100 U reverse transcriptase (Superscript II; Invitrogen Corp.), random hexamers and oligo dT. Real-time PCR was performed with a commercial kit (Fast Start DNA Master Sybr Green Kit (Roche Diagnostics) using a thermal cycler (Rotor-Gene 6000; Corbett Research, Mortlake, Australia). Data were analyzed with LightCycler software (Roche Diagnostics) and normalized either to TATA box binding protein (TBP), or to cyclophilin A housekeeping gene transcripts. Specific sense and anti-sense primers used for amplification are shown in Table 2.

**Table 2:** Primers used for reverse transcription quantitative PCR.

Name	Direction	Sequence 5'→3'
PLD1	Sense	GGTCAGAAAGATAACCCAGG
	Anti-sense	GAAGCGAGACAGCGAAATGG
Atrogin-1	Sense	CTCTGCCAGTACCACTTCTC
	Anti-sense	ATGGTCAGTGCCCCTCCAGG
LC3b	Sense	CTGGACAAGACCAAGTTCCT
	Anti-sense	AAGCCGTCTTCATCTCTCTC
S6K1	Sense	AGAGCACCTGCGTATGAATC
	Anti-sense	CACTGACTCTTTGAGACTGCC
Murf1	Sense	TGCATCTCCATGCTGGTGGC
	Anti-sense	CTTCTTCTCGTCCAGGATGG
Foxo1a	Sense	AGATCTACGAGTGGATGGTG
	Anti-sense	GGACAGATTGTGGCGAATTG
Foxo3a	Sense	GAGAGCAGATTTGGCAAAGG
	Anti-sense	CCTCATCTCCACACAGAACG
TBP	Sense	TGGTGTGCACAGGAGCCAAG
	Anti-sense	TTCACATCACAGCTCCCCAC
Cyclophilin A	Sense	ATGGCACTGGTGGCAAGTCC
	Anti-sense	TTGCCATTCCTGGACCCAAA

### *In vivo *experiments

Animals were treated in strict accordance with the guidelines of the Institutional Animal Care and Use Committee and to relevant national and European legislation, throughout the experiments.

Male BALB/c mice, 6 weeks old, were used (Charles River Laboratories Italia, Milan, Italy). Animals were housed in the animal facility under conventional conditions with constant temperature and humidity, and fed a standard diet. Tumor implantation was performed using cubic pieces of approximately 1 mm^3 ^of solid C26 tumor kept in liquid nitrogen. Tumor pieces were injected, using a trocar needle, under the dorsal skin of mice anesthetized with sodium pentobarbital 30 mg/kg. Control mice were injected with saline only. The animals were weighed every day. From day 8, a palpable tumor was visible.

Half of the control mice and half of mice with tumors were injected intraperitoneally with 0.1 mg/kg of a 2 mg/ml myriocin (Sigma-Aldrich) solution in 0.5% methanol. Control mice received 0.5% methanol in saline. Myriocin was injected until the end of the experiment. When the tumor-injected mice had lost 3 g of weight, they were killed by cervical dislocation. At the same time, a matched control (no tumor injection, same dose of myriocin) was also killed. The tumor-injected mice and matched controls were killed between days 13 and 17. The tibialis and gastrocnemius muscles were dissected from both hind limbs, weighed, and frozen in liquid N2-cooled isopentane and stored at -80°C for either histological or molecular analyses. Muscle sections (10 μm) were cut on a cryostat microtome, and stained with hematoxylin and eosin. Fiber CSAs (500 fibers per muscle) were measured using NIH ImageJ software.

### Statistical analyses

The statistical significance of data was assessed by ANOVA and Fisher tests, using StatView software version 5.0 (SAS Institute Inc., Cary, NC, USA).

## List of abbreviations

AVP: arginine-vasopressin; BSA: bovine serum albumin; CSA: cross-sectional area; DMEM: Dulbecco's modified Eagle's medium; IGF: insulin-like growth factor; MHC: myosin heavy chain; mTOR: mammalian target of rapamycin; OMS: 3-*O*-methylsphingomyelin; PLD: phospholipase D; PVDF: polyvinylidene fluoride; S1P: sphingosine-1-phosphate; TNF-α: tumor necrosis factor alpha.

## Competing interests

The authors declare that they have no competing interests.

## Authors' contributions

JDL and AZ performed most of the experiments, and analyzed the data. FS and AMI contributed to the *in vivo *experiments, KD contributed to the studies of protein metabolism, and MD contributed to the study of autophagy regulation. MP and DC supervised the analyses of sphingolipids. GN designed the research, analyzed the data, and wrote the article. FN, HV and EL participated in the coordination of the study and critically revised the manuscript. All the authors read and approved the final manuscript.

## Supplementary Material

Additional file 1 **Both tumor necrosis factor (TNF)-α and ceramide induce atrophy of C2C12 myotubes**. C2C12 myoblasts were differentiated in 2% horse serum-containing medium. The obtained myotubes were then treated with the various agents for 3 days, and then fixed and stained with periodic acid-Schiff technique [21]. The surface of randomly chosen 100 individual myotubes was measured using Scion Image Beta software (version 4.02; Scion Corporation, Frederick, MD, USA). **(A) **C2C12 myotubes were treated with various concentrations of mouse recombinant TNF-α, or 0.3 μg/ml cycloheximide used as a positive control. ***Different from control: *P *< 0.001. Representative of four experiments. **(B) **C2C12 myotubes were treated with various concentrations of cell-permeant C2, C6, or C8 ceramide. ***Different from control: *P *< 0.001. Representative of four experiments. Click here for file

Additional file 2 **GW4869 and 3-O-methylsphingomyelin, but not myriocin, prevent tumor necrosis factor (TNF)-α-induced atrophy of C2C12 myotubes**. C2C12 myotubes obtained as above were treated for 3 days with 1 ng/ml TNF-α, in the presence of various ceramide-synthesis inhibitors. They were fixed and immunolabelled for sarcomeric myosin heavy chain, and their surface was measured in 10 fields in each condition, using the method of Sultan *et al. *[21], with Image J software. **(A) **Effect of 100 nmol/l myriocin. ***Different from control: *P *< 0.001. Representative of four experiments. **(B) **Effect of 10 μmol/l GW4869, or 1 μmol/l 3-O-methylsphingomyelin (3-OMS). ***Different from control: *P *< 0.001; ++different from TNF-α alone: *P *< 0.01. Representative of four experiments. Click here for file

Additional file 3 **Tumor necrosis factor (TNF)-α and myriocin affect the amount of the Atrogin-1 target eIF3f (eukaryotic translation initiation factor 3 subunit f)**. L6 myotubes were treated for 3 days with or without TNF-α, in the presence of 100 nmol/l myriocin. The eIF3f protein level in cell extracts was analyzed by western blotting. Results were normalized to the amount of tubulin, and are the mean ± SE of four measurements. **+++**Different from control: *P *< 0.0001; *different from TNF- alone: *P *< 0.05,**P < 0.01. Click here for file

Additional file 4 **Lack of effect of ceramide-synthesis inhibition on the nuclear factor (NF)κB pathway**. L6 myotubes were treated for 10 minutes, or for the indicated time, with tumor necrosis factor (TNF)-α in the absence or presence of 100 nmol/l myriocin. Phospho-IKK-α/β (NFκB inhibitor kinase subunit-α/β) was analyzed by western blotting. The experiment shown is representative of two performed. Click here for file

Additional file 5 **Parameters of tandem mass spectrometry sphingolipid analyses. (A) **Physiological ceramide species: *m/z *mass of the [M+H- H2O]^+ ^ion for measurement in multiple reaction monitoring (MRM) mode. Product ion was *m/z *264.3. C16:0-D_31 _ceramide was used as internal standard. (**B) **Physiological sphingomyelin species: *m/z *mass of the [M-H]^- ^ion for measurement in MRM mode. Product ion was *m/z *79. C16:0-D_31 _sphingomyelin was used as internal standard. Click here for file
