# Supplementary figures and images for: TNF-α- and tumor-induced skeletal muscle atrophy involves sphingolipid metabolism
Source: Skelet Muscle. 2012 Jan 18;2:2. doi: 10.1186/2044-5040-2-2 (PMC3344678; doi:10.1186/2044-5040-2-2)

Additional file 1.

A

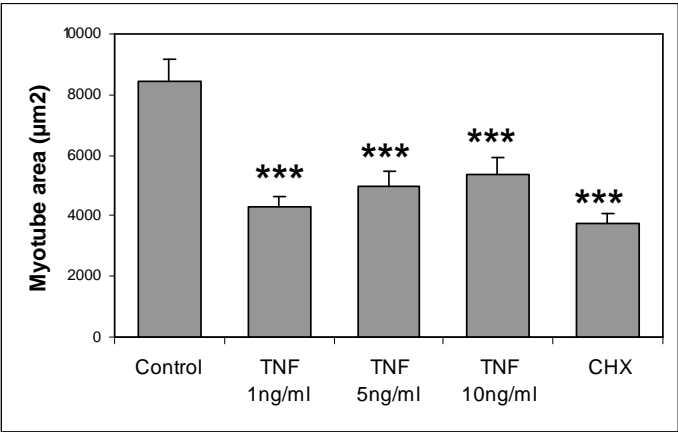

B

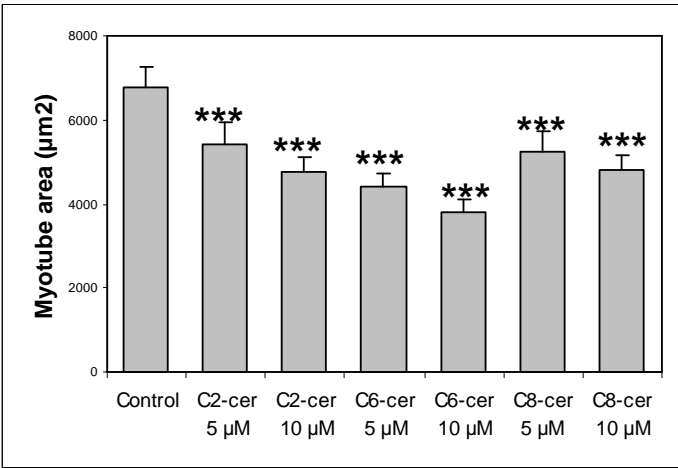

Supplement: Additional file 1 — Both tumor necrosis factor (TNF)-α and ceramide induce atrophy of C2C12 myotubes. C2C12 myoblasts were differentiated in 2% horse serum-containing medium. The obtained myotubes were then treated with the various agents for 3 days, and then fixed and stained with periodic acid-Schiff technique [21]. The surface of randomly chosen 100 individual myotubes was measured using Scion Image Beta software (version 4.02; Scion Corporation, Frederick, MD, USA). (A) C2C12 myotubes were treated with various concentrations of mouse recombinant TNF-α, or 0.3 μg/ml cycloheximide used as a positive control. ***Different from control: P < 0.001. Representative of four experiments. (B) C2C12 myotubes were treated with various concentrations of cell-permeant C2, C6, or C8 ceramide. ***Different from control: P < 0.001. Representative of four experiments. [file 2044-5040-2-2-S1.PDF]

Additional file 2 -

A

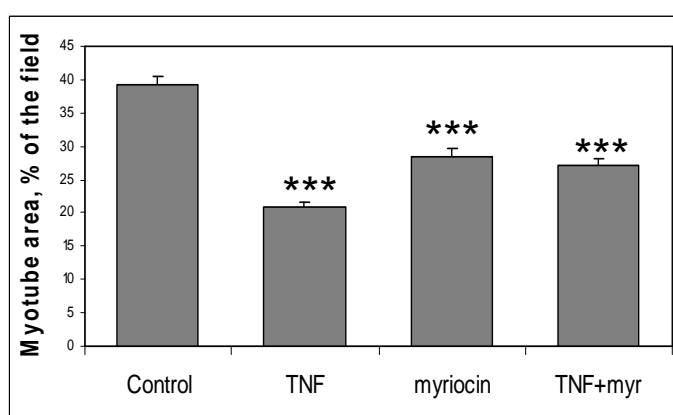

B

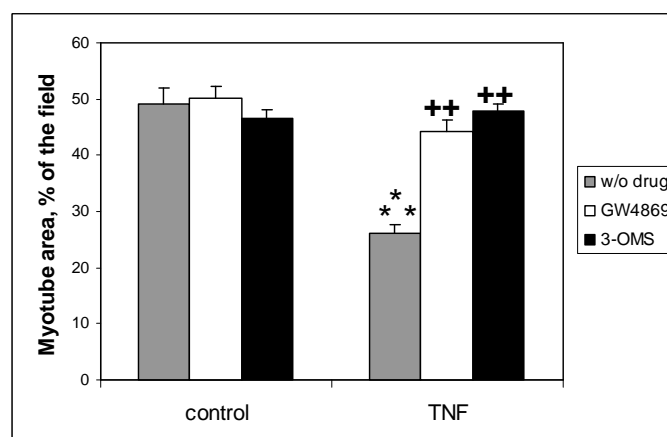

Supplement: Additional file 2 — GW4869 and 3-O-methylsphingomyelin, but not myriocin, prevent tumor necrosis factor (TNF)-α-induced atrophy of C2C12 myotubes. C2C12 myotubes obtained as above were treated for 3 days with 1 ng/ml TNF-α, in the presence of various ceramide-synthesis inhibitors. They were fixed and immunolabelled for sarcomeric myosin heavy chain, and their surface was measured in 10 fields in each condition, using the method of Sultan et al. [21], with Image J software. (A) Effect of 100 nmol/l myriocin. ***Different from control: P < 0.001. Representative of four experiments. (B) Effect of 10 μmol/l GW4869, or 1 μmol/l 3-O-methylsphingomyelin (3-OMS). ***Different from control: P < 0.001; ++different from TNF-α alone: P < 0.01. Representative of four experiments. [file 2044-5040-2-2-S2.PDF]

Additional file 3.

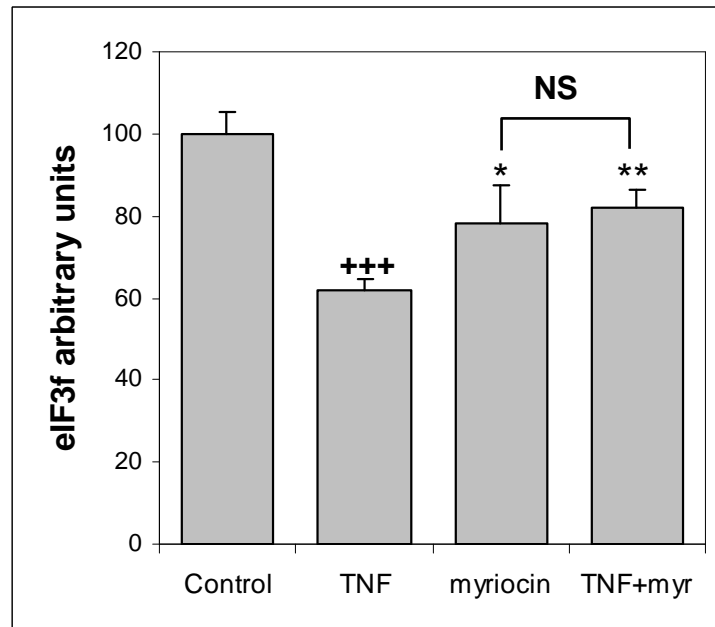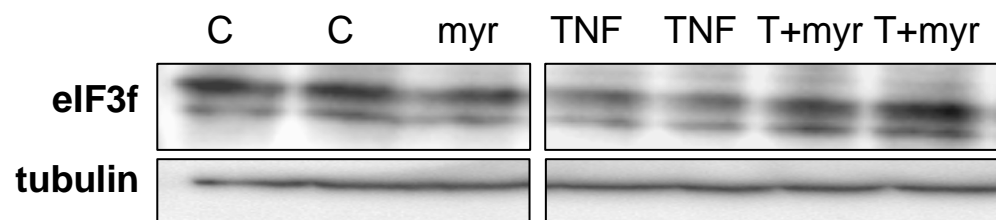

Supplement: Additional file 3 — Tumor necrosis factor (TNF)-α and myriocin affect the amount of the Atrogin-1 target eIF3f (eukaryotic translation initiation factor 3 subunit f). L6 myotubes were treated for 3 days with or without TNF-α, in the presence of 100 nmol/l myriocin. The eIF3f protein level in cell extracts was analyzed by western blotting. Results were normalized to the amount of tubulin, and are the mean ± SE of four measurements. +++Different from control: P < 0.0001; *different from TNF- alone: P < 0.05,**P < 0.01. [file 2044-5040-2-2-S3.PDF]

## Additional file 4 -

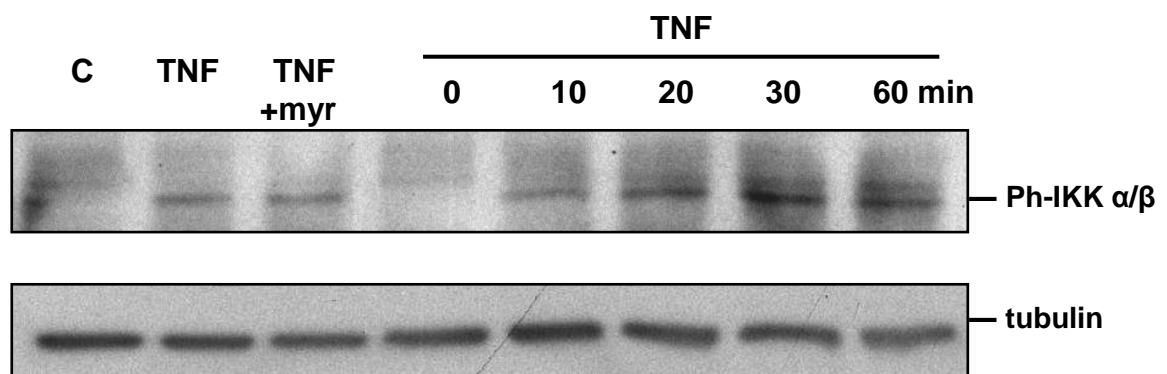

Supplement: Additional file 4 — Lack of effect of ceramide-synthesis inhibition on the nuclear factor (NF)κB pathway. L6 myotubes were treated for 10 minutes, or for the indicated time, with tumor necrosis factor (TNF)-α in the absence or presence of 100 nmol/l myriocin. Phospho-IKK-α/β (NFκB inhibitor kinase subunit-α/β) was analyzed by western blotting. The experiment shown is representative of two performed. [file 2044-5040-2-2-S4.PDF]
