# Supplementary material for: TNF-α- and tumor-induced skeletal muscle atrophy involves sphingolipid metabolism
Source: Skelet Muscle. 2012 Jan 18;2:2. doi: 10.1186/2044-5040-2-2 (PMC3344678; doi:10.1186/2044-5040-2-2)
Supplement: Additional file 5 — Parameters of tandem mass spectrometry sphingolipid analyses. (A) Physiological ceramide species: m/z mass of the [M+H- H2O]+ ion for measurement in multiple reaction monitoring (MRM) mode. Product ion was m/z 264.3. C16:0-D31 ceramide was used as internal standard. (B) Physiological sphingomyelin species: m/z mass of the [M-H]- ion for measurement in MRM mode. Product ion was m/z 79. C16:0-D31 sphingomyelin was used as internal standard. [file 2044-5040-2-2-S5.DOC]

**Additional file 5.**

**A**

| Fatty acid substituent | Molecular mass | *m/z* mass of the  [M+H-H2O]+ ion |
| --- | --- | --- |
| C16:2 | 533.7 | 516.7 |
| C16:1 | 535.7 | 518.7 |
| C16:0 | 537.7 | 520.7 |
| C16:2 OH | 549.7 | 532.7 |
| C16:1 OH | 551.7 | 534.7 |
| C16:0 OH | 553.7 | 536.7 |
| C18:2 | 561.7 | 544.7 |
| C18:1 | 563.7 | 546.7 |
| C18:0 | 565.7 | 548.7 |
| **C16:0-D_31_** | **568.7** | **551.7** |
| C18:2 OH | 577.7 | 560.7 |
| C18:1 OH | 579.7 | 562.7 |
| C18:0 OH | 581.7 | 564.7 |
| C20:2 | 589.7 | 572.7 |
| C20:1 | 591.7 | 574.7 |
| C20:0 | 593.7 | 576.7 |
| C20:2 OH | 605.7 | 588.7 |
| C20:1 OH | 607.7 | 590.7 |
| C20:0 OH | 609.7 | 592.7 |
| C22:2 | 617.7 | 600.7 |
| C22:1 | 619.7 | 602.7 |
| C22:0 | 621.7 | 604.7 |
| C22:2 OH | 633.7 | 616.7 |
| C23:0 or C22:1 OH | 635.7 | 618.7 |
| C22:0 OH | 637.7 | 620.7 |
| C24:2 | 645.7 | 628.7 |
| C24:1 | 647.7 | 630.7 |
| C24:0 | 649.7 | 632.7 |
| C24:2 OH | 661.7 | 644.7 |
| C25:0 or C24:1 OH | 663.7 | 646.7 |
| C24:0 OH | 665.7 | 648.7 |
| C26:2 | 673.7 | 656.7 |
| C26:1 | 675.7 | 658.7 |
| C26:0 | 677.7 | 660.7 |
| C26:2 OH | 689.7 | 672.7 |
| C26:1 OH | 691.7 | 674.7 |
| C26:0 OH | 693.7 | 676.7 |

**B**

| Fatty acid substituent | Molecular mass | *m/z* mass of the [M-H-CH2]- ion |
| --- | --- | --- |
| C16:2 | 698.8 | 683.8 |
| C16:1 | 700.8 | 685.8 |
| C16:0 | 702.8 | 687.8 |
| C16:2 OH | 714.8 | 699.8 |
| C16:1 OH | 716.8 | 701.8 |
| C16:0 OH | 718.8 | 703.8 |
| C18:2 | 726.8 | 711.8 |
| C18:1 | 728.8 | 713.8 |
| C18:0 | 730.8 | 715.8 |
| **C16:0-D_31_** | **733.8** | **718.8** |
| C18:2 OH | 742.8 | 727.8 |
| C18:1 OH | 744.8 | 729.8 |
| C18:0 OH | 746.8 | 731.8 |
| C20:2 | 754.8 | 739.8 |
| C20:1 | 756.8 | 741.8 |
| C20:0 | 758.8 | 743.8 |
| C20:2 OH | 770.8 | 755.8 |
| C20:1 OH | 772.8 | 757.8 |
| C20:0 OH | 774.8 | 759.8 |
| C22:2 | 782.8 | 767.8 |
| C22:1 | 784.8 | 769.8 |
| C22:0 | 786.8 | 771.8 |
| C22:2 OH | 798.8 | 783.8 |
| C23:0 or C22:1 OH | 800.8 | 785.8 |
| C22:0 OH | 802.8 | 787.8 |
| C24:2 | 810.8 | 795.8 |
| C24:1 | 812.8 | 797.8 |
| C24:0 | 814.8 | 799.8 |
| C24:2 OH | 826.8 | 811.8 |
| C25:0 or C24:1 OH | 828.8 | 813.8 |
| C24:0 OH | 830.8 | 815.8 |
| C26:2 | 838.8 | 823.8 |
| C26:1 | 840.8 | 825.8 |
| C26:0 | 842.8 | 827.8 |
| C26:2 OH | 854.8 | 839.8 |
| C26:1 OH | 856.8 | 841.8 |
| C26:0 OH | 858.8 | 843.8 |
